# The nicotinic acetylcholine receptor alpha 4 subunit contains a functionally relevant SNP Haplotype

**DOI:** 10.1186/s12863-015-0204-1

**Published:** 2015-05-02

**Authors:** Marlene Eggert, Georg Winterer, Mario Wanischeck, Jean-Charles Hoda, Daniel Bertrand, Ortrud Steinlein

**Affiliations:** Marlene Eggert, Institute of Human Genetics, Ludwig-Maximilians-University Hospital, 80336 Munich, Germany; Georg Winterer, Experimental and Clinical Research Center (ECRC), Charité – University Medicine Berlin, Berlin, Germany; Mario Wanischeck, Institute of Human Genetics, Ludwig-Maximilians-University Hospital, 80336 Munich, Germany; Jean-Charles Hoda, SwissCheckUp SA, 1400 Yverdon-Les-Bains, Switzerland; Daniel Bertrand, HiQScreen, 1222 Vésenaz, Geneva Switzerland; Ortrud K Steinlein, Institute of Human Genetics, Ludwig-Maximilians-University Hospital, 80336 Munich, Germany

**Keywords:** *CHRNA4*, Acetylcholine receptor, ACh sensitivity, Haplotype, mRNA stability

## Abstract

**Background:**

Non-coding single nucleotide polymorphisms within the nicotinic acetylcholine receptor alpha 4 subunit gene *(CHRNA4)* are robustly associated with various neurological and behavioral phenotypes including schizophrenia, cognition and smoking. The most commonly associated polymorphisms are located in exon 5 and segregate as part of a haplotype. So far it is unknown if this haplotype is indeed functional, or if the observed associations are an indirect effect caused by linkage disequilibrium with not yet identified adjacent functional variants. We therefore analyzed the functional relevance of the exon 5 haplotype alleles.

**Results:**

Using voltage clamp experiments we were able to show that the *CHRNA4* haplotype alleles differ with respect to their functional effects on receptor sensitivity including reversal of receptor sensitivity between low and high acetylcholine concentrations. The results indicate that underlying mechanisms might include differences in codon usage bias and changes in mRNA stability.

**Conclusions:**

Our data demonstrate that the complementary alleles of the *CHRNA4* exon 5 haplotype are functionally relevant, and might therefore be causative for the above mentioned associations.

**Electronic supplementary material:**

The online version of this article (doi:10.1186/s12863-015-0204-1) contains supplementary material, which is available to authorized users.

## Background

Cholinergic effects on cortical information processing and related cognitive performance are partly mediated through stimulation of high-affinity heteromeric α4β2 nicotinic acetylcholine receptors (nAChRs) [1-4]. α4β2 receptors are abundantly expressed in human cortex and hippocampus and possess high affinity to (partial) agonists including nicotine and varenicline [5-7]. Receptor upregulation occurs with chronic exposure to agonists and is thought to be regulated on the translational/posttranslational rather than transcriptional level [8-10].

In earlier work, we reported a causative relationship between mutations in exon 5 of *CHRNA4* (the nAChR α4-subunit coding gene) and the autosomal dominant nocturnal frontal lobe epilepsy (ADNFLE) - a rare seizure disorder that is frequently associated with neurocognitive deficits or psychiatric affections [11-14]. We then explored in a more recent study, whether association also might exist between human information processing and common *CHRNA4* exon 5 single nucleotide polymorphisms (SNPs). Using functional magnetic resonance imaging (fMRI), association, especially for SNP rs1044396, was observed with prefrontal/parietal information processing during a selective attention-requiring task [15]. Complementary behavioral, electrophysiological and neuroimaging studies from other groups have later provided converging evidence supporting the validity of this association. [16-25] Furthermore, we and others repeatedly observed that the common exon 5 SNPs are also associated with endophenotypes of nicotine dependence [26-29].

The *CHRNA4* SNPs that repeatedly showed association with neurological and behavioral traits all have in common that they are silent mutations, i.e. are not changing codons and therefore have no apparent effect on the protein sequence. However, during the last decade it has become obvious that not all silent SNPs are functionally neutral. In the present study, we therefore addressed the question if silent *CHRNA4* exon 5 SNPs are able to modulate mRNA or receptor properties. For this purpose we conducted experiments on receptor sensitivity and mRNA stability, and performed *in silico* analysis regarding possible codon usage differences introduced by the haplotype alleles.

## Results

### Receptor sensitivity analysis

Exon 5 of the *CHRNA4* gene contains a linkage group (haplotype) of synonymous variants (haplotype 5’-rs1044393, rs1044394, rs2229959, rs2229960, rs1044396, rs1044397-3’) of hitherto unknown functional relevance. This haplotype rather than single SNPs was chosen for the here reported functional studies because exon 5 SNPs are in linkage disequilibrium with each other. It is therefore not possible to decide if, for example, associations found for a specific SNP are indeed caused by this SNP or by another one located on the same haplotype. Analysis of the haplotype therefore allowed us to simultaneously include all major exon 5 SNPs into our search for functional effects.

Haplotype allele hap1 (T-T-G-T-C-G) corresponds to the NCBI reference sequence (NM000744.5) and has a frequency of 9% in the general population, while the complementary hap2 allele (C-C-T-C-T-A) accounts for 52% of all alleles (according to our reference student population). Hap 1 and Hap 2 differ in each single SNP position. Besides hap1 and hap2 at least six other alleles of this haplotype are present in the normal population. These haplotypes share one or more SNP allele with the major haplotypes Hap1 and Hap2 and can therefore be expected to produce intermediate results in functional analysis. They were therefore not included in the present study.

**Figure 1:**
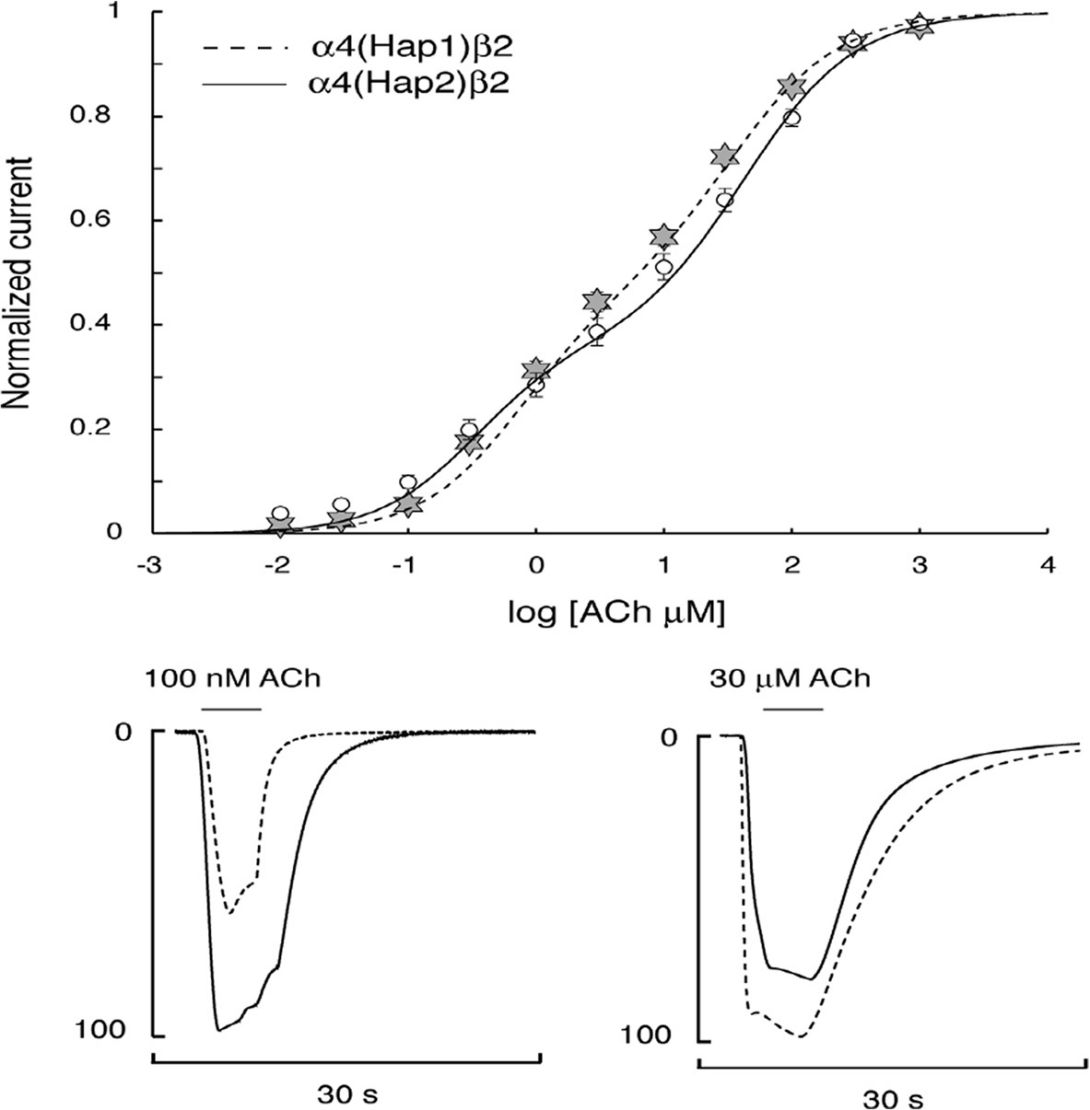
Graphical representation of electrophysiological experiments. **a)** Concentration activation curves for the α4(hap1)β2 and α4(hap2)β2 receptors. To minimize scatter, data were collected from large batches of cells. Data obtained from 70 cells expressing the α4(hap1)β2 are indicated by stars whereas circles correspond to results collected for 65 cells expressing the α4(hap2)β2. Bars indicate the SEM. Continuous curve is the best fit obtained with the sum of two Hill equations for the α4(hap2)β2 and dashed line for the α4(hap1)β2. Representative currents evoked by 100 nM and 30 μM ACh are illustrated in the lower panel. For comparison responses have been normalized to 100% for the largest evoked current. These data illustrate the differential, i.e. inverse, sensitivity at the two concentrations between the two haplotype alleles of the α4β2 receptor.

Heterologous expression experiments showed that α4β2-receptors with both haplotypes yield functional receptors with current amplitudes that increased in a dose-dependent manner with the ACh concentrations (Figure 1). For low ACh doses the currents from hap2 receptors (incl. rs1044396 T-allele) were up to 130% larger than those from hap1 receptors (incl. rs1044396 C-allele), resulting in a shift of the hα4(hap2)β2 curve towards lower concentrations with respect to the curve for hα4(hap1)β2. For higher doses of ACh the opposite effect was found with currents for hα4(hap2)β2 that were about 13% lower than those obtained for hα4(hap1)β2. The EC50L for hα4(hap2)β2 (0.33 μM ± 0.017) differed significantly (P ≤ 0.001, n = 65) from hα4(hap1)β2 (0.72 ± 0.04), indicating a higher sensitivity to ACh for hα4(hap2)β2 at low ACh concentrations. Interestingly, the EC50H data demonstrate a switch in this behavior, pointing to a lower sensitivity to ACh for hα4(hap2)β2 (42.5 μM ± 3) compared to hα4(hap1)β2 (33.6 μM ± 2.3, P ≤ 0.05, n = 65) in case of high ACh concentrations (Figure 1).

### mRNA stability analysis

When comparing the mRNA decay time difference of hap 1 and 2, none of the time differences for the four upstream fragments were significant. However, regarding the time difference 0 to 24 hrs for the most downstream fragment, our results revealed that the 3’ end of hap 1 mRNA was significantly more slowly degraded than that of hap 2 (P = 0.03) (Table 1).

**Table 1:** **Delta C**_**q**_ **mean values and p-values for qPCR fragments 1 to 5**

**mRNA decay time difference**	**qPCR fragment**														
	**1**			**2**			**3**			**4**			**5**		
	**Δ C**_**q**_ **mean**		**p-value**	**Δ C**_**q**_ **mean**		**p-value**	**Δ C**_**q**_ **mean**		**p-value**	**Δ C**_**q**_ **mean**		**p-value**	**Δ C**_**q**_ **mean**		**p-value**
	**hap 1**	**hap 2**	**hap1/2**	**hap 1**	**hap 2**	**hap1/2**	**hap 1**	**hap 2**	**hap1/2**	**hap 1**	**hap 2**	**hap1/2**	**hap 1**	**hap 2**	**hap1/2**
0 to 3 hrs	1.16	0.29	0.242	1.91	0.09	0.029^1^	1.61	0.26	0.066	1.07	-0.07	0.08	1.17	0.37	0.436
	(-0.72 - 3.04)	(-1.57 -2.15)		(-0.31 -4.12)	(-1.77 -1.94)		(-0.73 -3.95)	(-1.61 -2.13)		(-0.70 -2.83)	(-1.66 -1.51)		(-1.48 -3.81)	(-1.73 -2.47)	
0 to 6 hrs	2.54	1.95	0.403	3.34	2.41	0.313	3.04	2.48	0.546	2.23	1.7	0.503	3.69	3.34	0.821
	(0.49-4.58)	(0.63-3.27)		(0.79-5.89)	(0.77-4.04)		(0.43-5.65)	(0.75-4.21)		(0.26-4.19)	(0.29-3.11)		(0.72-6.66)	(0.71-5.97)	
0 to 24 hrs	5.74	5.64	0.918	6.18	6.24	0.952	5.06	4.77	0.778	5.21	4.77	0.679	8.16	10.73	0.036^2^
	(3.86-7.62)	(3.22-8.06)		(4.02-8.33)	(3.84-8.63)		(2.79-7.34)	(2.55-7.00)		(3.55-6.87)	(2.23-7.32)		(5.42-10.90)	(6.03-13.44)	

Δ C_q_ mean, delta C_q_ mean: difference of the mean quantification cycle; confidence intervals are given in brackets.

^1^confidence interval for delta C_q_-mean values of hap1 and hap2 are not significant.

^2^confidence interval for delta C_q_-mean values of hap1 and hap2 are significant.

### mRNA secondary structure prediction and codon usage analysis

Analysis of the predicted mRNA secondary structure showed marked differences between the two haplotype alleles (see Additional file 1: Figure S1). Codon usage analysis showed that most SNPs introduced changes from frequently to more rarely used codons or *vise versa*. These effects were most pronounced for rs2229959, rs1044396 and rs1044397 (see Table 2).

**Table 2:** **Changes of codon usage frequencies within the haplotype**

**dbSNP ID**	**amino acid positions**	**Alleles (hap 1/hap 2)**	**Codon usage frequencies (hap 1/hap 2)**
rs1044393	D213	GAT/GAC	21,8/25,1
rs1044394	C226	TGT/TGC	10,6/12,6
rs2229959	P403	CCG/CCT	6,9/17,5
rs2229960	C409	TGT/TGC	10,6/12,6
rs1044396	S543	AGC/AGT	19,5/12,1
rs1044397	A553	G/A	7,4/15,8

Data source for codon usage: http://www.kazusa.or.jp/codon/.

## Discussion

Taken together, our experimental data show that the *CHRNA4* haplotype alleles exert different functional effects on mRNA stability as well as on receptor sensitivity including reversal of receptor sensitivity between low and high ACh concentrations. Furthermore, i*n silico* analysis predict that the haplotype alleles also differ with respect to codon usage and mRNA secondary structure. The experiments were conducted using clones that contained identical fragments from the *CHRNA4* coding region, the only differences between the clones being the respective alleles of the five SNPs composing the haplotype. Thus the variation observed in both ACh sensitivity and mRNA stability should be attributable to the SNPs within the haplotype. Our results therefore strongly suggest that one or more of the synonymous SNPs that constitute the haplotype are functionally relevant. Such a conclusion would not be too surprising, given that several examples exist in which silent SNPs have been found to modulate gene function, for example by altering mRNA stability, translation efficiency or protein conformation [30-32].

Various mechanisms could explain the distinctive dose-response curves of the two haplotypes. It is a possibility that the observed changes in mRNA stability are one of the mechanisms that contribute to the haplotype-dependent differences in ACh sensitivity. One explanation could be that the altered mRNA stability may lead to an increased translation rate of *CHRNA4* mRNA carrying the more stable haplotype 1. Such an increased translation rate would expand the amount of α4 subunit protein in hap 1 carriers while the amount of β2 subunit protein would remain constant. This in turn could alter the nAChRs stoichiometry so that more (α4)_3_(β2)_2_ than (α4)_2_(β2)_3_ receptor subtypes are assembled. Such changes in stoichiometry are a factor known to influence several functional receptor characteristics and to increase receptor affinity [33,34]. It is also possible that, apart from mRNA stability, additional mechanisms are responsible for the observed differences in agonist sensitivity. For example, changes in the mRNA sequence are known to affect its folding which in turn can influence the efficiency and speed of protein synthesis [35]. This mechanism would also be able to affect the ratio of α4 versus β2 subunits within the mature nAChR. Furthermore, codon bias is discussed as mechanism for a gene expression regulation because it has been observed that genes with lower expression levels prefer codons which are recognized by tRNAs with lower gene copy numbers [36].

Another factor that might play a role would be codon bias at the ribosome. It is assumed that the speed at which a given mRNA is decoded at the ribosomes largely depends on the availability of individual tRNA molecules. However, most amino acids can be encoded by more than one base pair triplet, and there are significant differences with respect to the frequency with which individual codons occur in genes. In fact, synonymous codons are used at nonrandom frequencies, a phenomenon termed codon usage bias. Such differences in codon usage are not only found between species, but in some examples also have been described for different tissues from the same individual [37]. Both codon usage and tRNA gene numbers evolved together, and, consequently, tRNAs that recognize frequently used codons are usually more abundant at the ribosome and are therefore more readily available for translation [37,38]. Thus a silent SNP changing a frequently used codon into a rarer one can slow down the speed of mRNA translation. In fact, synonymous codon usage is recognized as the primary cause of non-uniform translation rates, a mechanism known to cause for differential maturation and folding of nascent polypeptides [39]. These differences in polypeptide processing are possible because the time newly synthesized polypeptides spend at the ribosome is used to introduce various modifications that are important for protein folding, stability, and interaction with binding partners [40]. The silent SNPs that constitute the *CHRNA4* haplotype introduce several changes from frequent to rarer used codons (or *vise versa*, see Table 2) and could therefore alter, by the above discussed mechanisms, functional characteristics of the nAChR such as stoichiometry, surface expression or function. Pathomechanisms like this have already been reported for other silent SNPs associated with human disorders [31,35,41].

It appears remarkable that, within our experimental setting, mRNA stability was only altered in the downstream (3’) region of the cloned *CHRNA4* coding region fragment. These observations suggest that it is the SNPs in the 3’ part of the haplotype that are able to alter mRNA stability. Interestingly, the 3’ end of the *CHRNA4* haplotype harbors the two silent SNPs that have most consistently shown association with clinical phenotypes. For example, recent work from several groups including our own reported association between rs1044396/rs1044397 and endophenotypes of schizophrenia as well as nicotine addiction. Both SNPs are significantly associated with cognitive endophenotypes such as brain activation (N100-amplitude – in particular in prefrontal cortex) during selective-attention-requiring tasks [15-19,21,23-26]. With a minor allele frequency above 0.45 both SNPs would be common enough to contribute considerably to the inter-individual variability in the processing of cognitive tasks, addictive behavior and psychiatric disorders within the general population. Additional studies are needed to shed light on the complex interactions between these silent nAChR variants, differences in nAChR function, and the inter-individual variability of neurological and behavioral traits in humans.

## Conclusions

Our experimental and *in silico* data demonstrate that the complementary alleles of the *CHRNA4* exon 5 haplotype differ with respect to mRNA stability, codon usage, and agonist sensitivity. These results render it possible that one or more of the haplotype-constituting SNPs are causative for the previously reported associations with neurological and behavioral phenotypes.

## Materials and Methods

### Receptor sensitivity analysis

The cDNAs with either one of the two complementary *CHRNA4* haplotypes and with the *CHRNB2* wild type sequence were injected in *Xenopus* oocytes in equal amounts and the electrophysiological properties of the α4β2 nAChR channel were determined using a two-electrode voltage clamp technique (HiClamp, Multichannel System®, Reutlingen Germany) and applying different concentrations of acetylcholine (ACh). Concentration-activation curves were fitted using a Hill equation in the form Y = 1/1 + (EC50/ x)^ nH(1) where: y = the fraction of evoked current, EC50 = concentration for 50% activation of the high affinity, nH = the apparent cooperativity for the high affinity, x = agonist concentration. Concentration-inhibition curves are fit with a comparable equation Y = 1/1 + (x/IC50)^nH(2) where: y = the fraction of remaining current, IC50 = concentration for 50% inhibition, nH = the apparent cooperativity, x = antagonist concentration.

### mRNA stability testing

**Figure 2:**
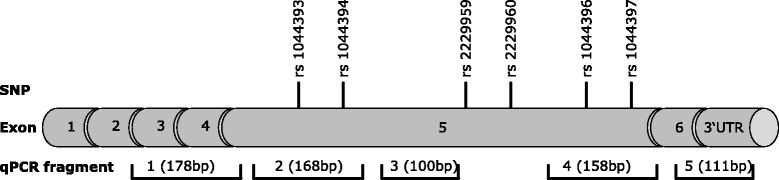
Schematic representation of the *CHRNA4* gene. The positions of SNPs that constitute the *CHRNA4* haplotype (see main text) are indicated above, the fragments used for mRNA stability testing below the transcript.

The Tet-Off® advanced inducible gene expression system was purchased from Clontech (Saint-Germain-en-Laye, France). The coding sequence of *CHRNA4* hap 1, respectively hap 2 was obtained by PCR amplification of human DNA. After KpnI and EcorV digestion of the pTRE-Tight-BI-AcGFP1 vector, the coding sequence of *CHRNA4* hap 1, respectively hap 2 (1884 nt) was ligated into the multiple cloning site of pTRE-Tight-BI-AcGFP1 downstream of the doxycycline-dependent promoter. The resulting construct had the following structure (origin of fragments given in brackets): (pTRE-Tight) …GCTCGGTAC (pTRACER) CGAGCTCGGATCCA (*CHRNA4* uncoding) CTAGTAGTGCGCC (*CHRNA4* coding) ATG…TAG (*CHRNA4* uncoding) GAATAG (pTRACER) GAATTCTGCAGAT (pTRE-Tight) ATCTC… After cloning the inserts were confirmed by sequencing. Culturing of Tet-Off® human embryonic kidney cells (HEK) 293 (Clontech, Saint-Germain-en-Laye, France) was performed using standard protocols. HEK 293 cells were transfected with 10 ng plasmid pTRE-Tight-BI-AcGFP1 containing the coding sequence of *CHRNA4* haplotype 1 and 2, respectively, using 3 μl of TransIT ®-LT1 Transfection Reagent (MoBiTec GmbH, Göttingen, Germany) with 24 h transfection prior to medium change and addition of 1 μg doxycycline. RNA was extracted after 0; 3; 6 and 24 hours of doxycycline incubation by using QIAshredder and RNeasy kit, including DNase treatment of 10 min in solution, according to the manufacturer’s protocol (Qiagen, Hilden, Germany). Real-time PCR was performed targeting five fragments of the *CHRNA4* coding sequence (primer sequences: 1 F GCTCATTGACGTGGATGAGA, 1R CCCGTCAGCATTGTTGTAGA, 2 F GCTGGACTTCTGGGAGAGTG, 2R AGGGGATGATGAGGTTGATG, 3 F TGCTCATCACCGAGATCATC, 3R ATGACGATGGACAGGGTGAC, 5 F AAGGAGCCCTCTTCGGTGTC, 5R CTTCGGCCTTCAGGTGGTCT, 5 F GGCTGGCATGATCTAGGAAT, 5R GGGAGGTGTGGGAGGTTTTT, AcGFP_F ATGATGTATCGCCCTCGAAC, AcGFP R CACATGAAGCAGCACGACTT) (Figure 2). Amplification efficiency and test linearity (correlation coefficient R2) were assessed for each primer pair. The reactions were carried out in the Mini Opticon CFD-3120 cycler (Bio-Rad, Munich, Germany). All experiments were repeated independently three times with triplicate biological and triplicate technical samples (nine experiments each in total). Statistical analysis was performed with program R to compare the haplotype 1 and 2 RNA degradation rate for each target fragment. A p value of p < 0.05 was considered statistically significant.

### Prediction of mRNA secondary structure and codon usage

Changes in the minimum free energy (MFE) secondary structure caused by the haplotype alleles were predicted by the use of the RNA fold web server, Vienna RNA package (http://rna.tbi.univie.ac.at/cgi-bin/RNAfold.cgi). A prediction software was employed for the codon usage analysis (http://www.kazusa.or.jp/codon).

## Additional file

Additional file 1: Figure S1. Minimum free energy structure of CHRNA4 haplotype mRNAs. The minimum free energy secondary structures for both CHRNA4 haplotypes were calculated from base-pairing probabilities, using the share ware program RNAfold web server (http://rna.tbi.univie.ac.at/cgi-bin/RNAfold.cgi). The prediction shows the optimal secondary structure [42].

## Abbreviations

ACh: Acetylcholine
ADNFLE: Autosomal dominant nocturnal frontal lobe epilepsy
*CHRNA4*: Nicotinic acetylcholine receptor alpha 4 subunit gene
*CHRNB2*: Nicotinic acetylcholine receptor beta 2 subunit gene
fMRI: Functional magnetic resonance imaging
nAChRs: Nicotinic acetylcholine receptors
SNPs: Single nucleotide polymorphisms
