# Supplementary figures and images for: The nicotinic acetylcholine receptor alpha 4 subunit contains a functionally relevant SNP Haplotype
Source: BMC Genet. 2015 May 2;16:46. doi: 10.1186/s12863-015-0204-1 (PMC4417232; doi:10.1186/s12863-015-0204-1)

## Slide 1
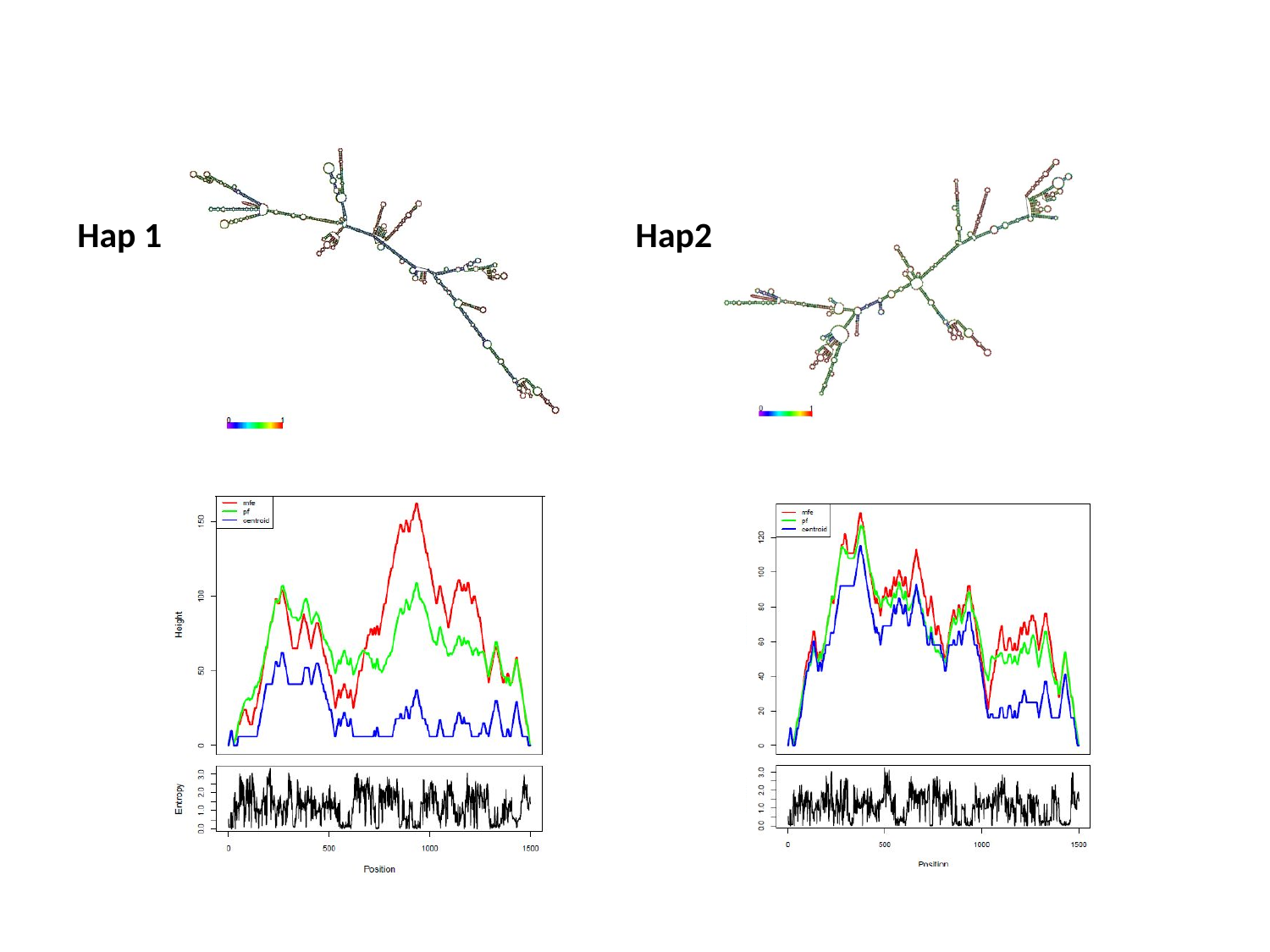

Hap 1 Hap2

Supplement: Additional file 1: Figure S1. — Minimum free energy structure of CHRNA4 haplotype mRNAs. The minimum free energy secondary structures for both CHRNA4 haplotypes were calculated from base-pairing probabilities, using the share ware program RNAfold web server (http://rna.tbi.univie.ac.at/cgi-bin/RNAfold.cgi). The prediction shows the optimal secondary structure [42]. [file 12863_2015_204_MOESM1_ESM.pptx]
